# Unusual Site for a White Nodule on the Palatine Tonsil: Presentation, Differential Diagnosis, and Discussion

**DOI:** 10.1155/2021/1371329

**Published:** 2021-07-08

**Authors:** Ashwag Yagoub Aloyouny

**Affiliations:** Basic Dental Sciences Department, College of Dentistry, Princess Nourah Bint Abdulrahman University, Riyadh, Saudi Arabia

## Abstract

**Introduction:**

Palatine tonsils are part of the mucosa-associated lymphoid tissue, located in the oropharyngeal region. Although these tissues protect the body from foreign intruders, they are more prone to infections due to their anatomical structure and location. For instance, the differential diagnosis of a white lesion on the palatine tonsil can range from benign to malignant lesions. Oral lymphoepithelial cysts commonly arise as painless, yellowish nodules on the floor of the mouth and the ventral or lateral surface of the tongue. *Case Presentation*. This paper presents a rare case of an unusual site of a lymphoepithelial cyst (LEC) in the oral cavity. The lesion was located in the tonsil of a 20-year-old woman with a chief complaint of a painless, white lump in the back of the mouth for nine months. *Discussion*. The differential diagnosis of a white lesion on the palatine tonsil is caused by several factors, such as bacterial, viral, and fungal infections; trauma; stones; cysts; abscess; or cancer. In this case, both the clinical presentation and extra- and intraoral examinations were highly associated with LEC. Oral LEC etiopathogenesis is uncertain, and several theories have been proposed to discuss the causes of LEC. In addition, oral LEC could be monitored without surgical intervention if the nodule is asymptomatic.

**Conclusion:**

We emphasize the importance of a thorough clinical examination of oral and oropharyngeal lesions, which are usually neglected.

## 1. Introduction

The palatine tonsil is a component of the posterior oropharynx lymphoid tissue ring called Waldeyer's ring. The location of the palatine tonsil makes it more amenable to bacterial infections and cyst formation [1].

The differential diagnosis of a white lesion on the palatine tonsils encompasses benign and malignant lesions. Benign lesions are more common in this location, including oral lymphoepithelial cysts (LECs), tonsilloliths, and lipomas and infectious entities such as tonsillitis, strep throat, infectious mononucleosis, oropharyngeal candidiasis, and peritonsillar abscess [2]. It could also include benign lesions that have a potential for malignant transformation such as papilloma. Moreover, although malignant lesions rarely arise from the palatine tonsils, they are not excluded from the differential diagnosis list [3].

LECs are rare, benign, developmental, and nonodontogenic cysts. They may develop in any area of the body, which consists of lymphoid tissues. Usually, they are less than 1 cm in size. Oral LEC has a high occurrence on the floor of the mouth and in the lateral and ventral tongue. It affects females more than males (2 : 1) at any age [2].

The phenomenon of oral LECs in the palatine tonsils is very unlikely. Additionally, very few reported cases in the literature present oral LECs in the tonsils. Herein, we present a rare case of an oral LEC that appeared in the left palatine tonsil and discuss its resolution without intervention.

The work has been reported in line with the SCARE criteria [4].

## 2. Case Report

**Figure 1:**
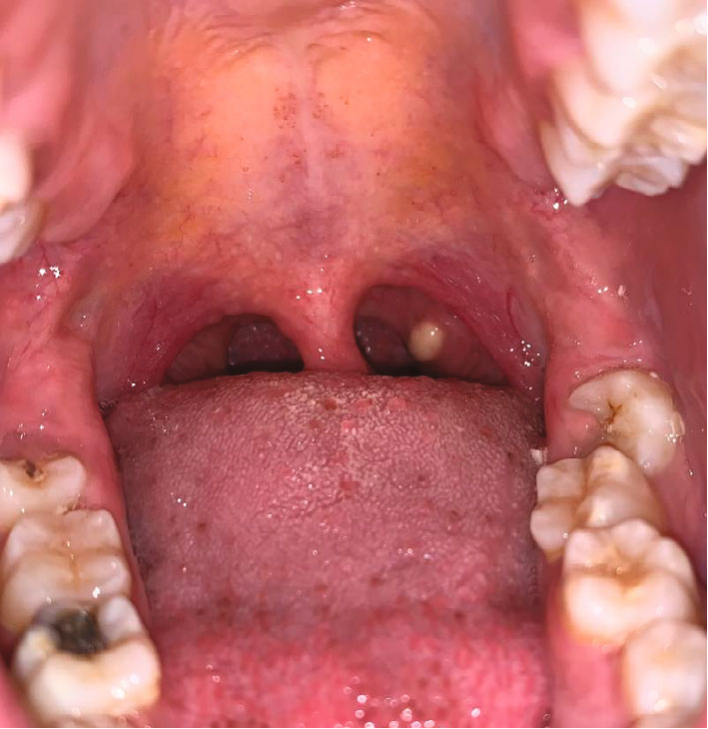
Clinical presentation of a 0.5 cm, yellow nodule with intact mucosa arising from the left palatine tonsil.

A 20-year-old woman visited the oral medicine clinic for evaluation of a nine-month history of a painless, white lesion at the back of the mouth. She believed that the lesion started as a pinhead size and then grew in size over time until it reached the present size. She did not recall any trauma or surgery in the area of the white patch. Her medical and dental histories were noncontributory. She denied taking any over-the-counter or prescribed medications, such as antibiotics or steroids. In addition, genetic problems, family medical history, and social history were unremarkable. Furthermore, the lesion was worrisome, which had a negative impact on daily life. Extraoral clinical examination was not significant. Routine laboratory blood test results were normal. The patient denied a history of fever, fatigue, body rashes, stuffy nose, headache, earaches, stiff neck, hoarseness, sore throat, coughing, sneezing, difficulty chewing or swallowing, halitosis, lymphadenopathy, and difficulty breathing. She also denied having hemoptysis and an unexplained weight loss in the last few weeks. Intraoral inspection revealed a 0.5 cm, asymptomatic, light yellow-white hued, smooth, raised, soft on palpation, movable, well-circumscribed, unilateral, localized submucosal nodule, exhibiting no exudate arising from the normal-sized left palatine tonsil (Figure 1). Radiographic interpretation of orthopantomograph showed normal anatomical structures and no signs of radiopacities in the left palatine tonsil region.

The differential diagnoses of the white lesion on the palatine tonsil, in this reported case, included tonsillar cyst, tonsilloliths, tonsillitis, papilloma, lipoma, strep throat, infectious mononucleosis, oropharyngeal candidiasis, and peritonsillar abscess.

In this case, the distinct clinical appearance, features, and clinical examination highly indicated an LEC. The patient was assured that the clinical appearance of the white lump in the tonsil was highly associated with the diagnosis of a benign nodule called an oral lymphoepithelial cyst. She was also provided with information regarding the possible treatment options and associated outcomes.

**Figure 2:**
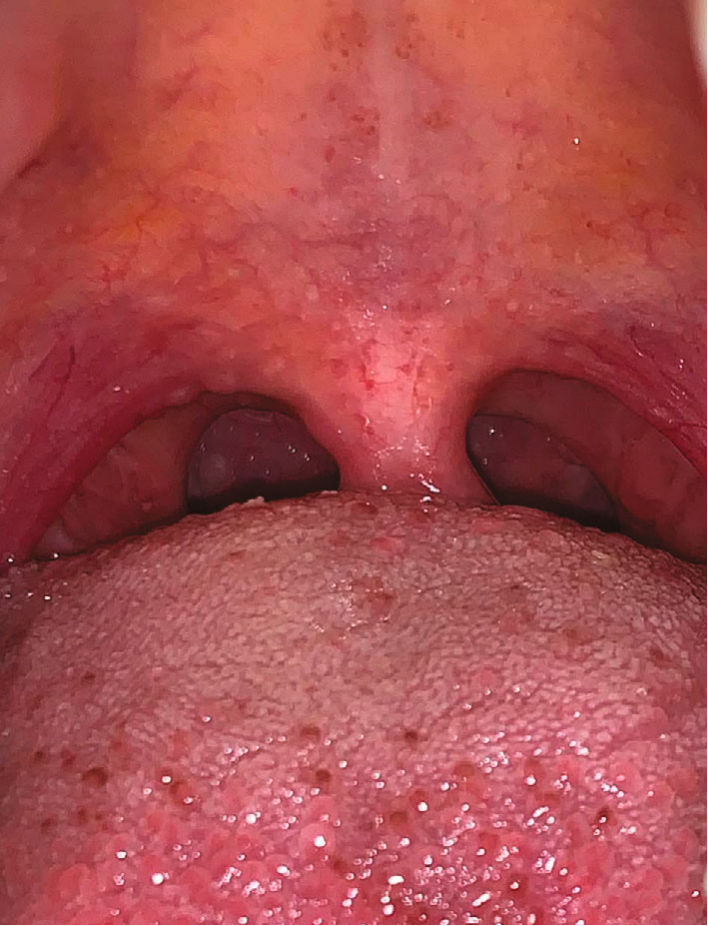
At eight-month follow-up visit, the yellow nodule in the left palatine tonsil completely disappeared.

The ideal management for the white lesion on the palatine tonsil is to excise the lesion along with the whole tonsil. Therefore, an oral medicine specialist recommended performing a left palatine tonsillectomy. However, the surgical procedure of the affected palatine tonsil was not an option from the patient's side. Notably, the patient was anxious and worried about the surgical procedure and its consequences. Therefore, she refused to proceed with the surgical option. Because the nodule was asymptomatic and based on the lesion size (5 mm) and the patient's decision, the lesion was monitored regularly for an eight-month period. One-month, two-month, three-month, six-month, and eight-month follow-up periods were scheduled to monitor the lesion. The nodule did not change in size or color during this period. Surprisingly, the lesion ruptured and resolved spontaneously without any conventional or surgical intervention (Figure 2). At the one- and two-year follow-up, there were no signs of lesion recurrence.

## 3. Discussion

The posterior oropharynx contains a lymphoid tissue ring called Waldeyer's ring, which mainly involves the palatine tonsils, pharyngeal adenoid tonsils, Eustachian tonsils, and lingual tonsils. These lymphoid tonsils have special anatomical structures and locations, which make them more susceptible to bacterial infections and cyst formation [1].

The differential diagnosis of a white lesion on the palatine tonsil includes tonsillar cysts or oral LECs, tonsilloliths, papillomas, lipomas, tonsillitis, strep throat or streptococcal pharyngitis, infectious mononucleosis, peritonsillar abscess, oropharyngeal candidiasis, and palatine cancers [5].

Tonsilloliths or tonsil stones are unilateral, painful, and hard on palpation and consist of calcium deposits in the lymphoid tissue crypts. These stones occur due to the accumulation of mucus, bacteria, and food particles, which are accompanied by dysphagia, halitosis, and otalgia [6]. Papilloma is not uncommon, benign, exophytic, cauliflower-like verrucous lesion that is characterized by a unique appearance and possesses finger-like projections on the rough surface of the lesion [7]. Lipomas are very rare to develop in the palatine tonsil, and there are few documented cases in the English literature [8].

Bacterial infections of the palatine tonsils, such as tonsillitis and strep throat or streptococcal pharyngitis, are contagious and usually caused by *Streptococcus pyogenes*. Bacterial infections commonly occur along with many symptoms such as fever, fatigue, sore throat, headache, and difficulty swallowing. If left untreated, it could progress to a peritonsillar abscess. Viral infections of the palatine tonsils, such as infectious mononucleosis or mono, are characterized by bilateral, white-yellowish pus covering both swollen tonsils. Infectious mononucleosis is caused by a contagious virus called the Epstein-Barr virus. Bacterial and viral infections commonly occur along with many symptoms such as fever, fatigue, sore throat, headaches, and difficulty swallowing [9].

Patients with a suppressed body immune system are at a high risk of oropharyngeal candidiasis. It is the most common type of fungal infection in the oral cavity and is usually caused by *Candida albicans*. Low body immunity could be caused by uncontrolled diabetes, long-term use of antibiotics, or steroid medications. The diagnosis of the infection can be confirmed by collecting throat swab samples and culture from the site of the white patches or lesions [9].

In our case, the patient was in a very good health condition and did not complain of any abnormal signs or symptoms; the nodule had a distinct clinical appearance and was highly associated with LEC. LEC is an uncommon developmental, nonodontogenic cyst that was first described as a branchial cleft cyst in 1962 by Gold [10].

Yang et al. [2] studied the most common sites of oral LECs out of 120 reported cases. The study illustrated that 70.7% of oral LECs were encountered on the floor of the mouth, followed by the lateral surface (10.7%) and then the ventral surface of the tongue (7.3%). This study did not report any cases of palatine tonsils. Sykara et al. [11] collected data from 26 published cases, only two of which were in the palatine tonsils.

The pathogenesis of oral LEC is controversial, and many theories have been proposed to discuss the etiology of LEC. However, the most accepted theory was proposed by Knapp. He believed that oral LECs are pseudocysts that develop from submucosal lymphoid tissue aggregates on the floor of the mouth, ventral tongue, and soft palate [12].

Histopathological evaluation of oral LEC demonstrates a thick cystic cavity composed of parakeratinized stratified squamous epithelial lining filled with viscous fluid and desquamated keratin. The cystic wall is infiltrated by lymphocytes organized as germinal centers [13].

Oral LEC can be managed either conservatively or surgically based on the medical history, lesion size, lesion location, and patient discomfort. For instance, patients who have asymptomatic, small oral LECs can be monitored periodically. These small-sized oral LECs may rupture and disappear without surgical procedures. In the present case, the lesion was painless, approximately 0.5 cm in its greatest dimension, and the patient refused any intervention, but follow-up visits were conducted to monitor the lesion. The lesion disappeared after eight months of periodic follow-up visits [14]. Furthermore, for patients who complain of a large-sized lesion that causes dysphagia or risk of airway obstruction, surgical removal is indicated to avoid life-threatening conditions and improve patient quality of life.

## 4. Conclusion

The incidence of oral LEC in the palatine tonsils is uncommon. Based on the lesion size and the symptoms reported by the patient, oral LEC may be managed only with follow-up and clinical intraoral examination. In addition, oral LECs can resolve spontaneously without any kind of intervention.

## 5. Patient Perspective

The patient was assured that the lesion was benign and small in size and could resolve without intervention. The resolution of the lesion improved her self-confidence.
